# A new Critical Care channel in *F1000Research*

**DOI:** 10.12688/f1000research.7398.1

**Published:** 2015-11-19

**Authors:** Jean-Louis Vincent

**Affiliations:** 1 Department of Intensive Care, Erasme Hospital, Université libre de Bruxelles, Brussels, route de Lennik 808, 1070, Belgium

**Keywords:** Critical care

## Abstract

A new channel for Critical Care offers clinicians and medical researchers a platform for publishing new research without the barriers and delays they often encounter in traditional journals. The channel welcomes research and debate across the broad field of acute care and emergency medicine, including confirmatory and negative/null studies supported by new data

Critical care medicine is a rapidly expanding hospital speciality, taking an increasingly important place at the centre of acute hospital care ^1^. A vast amount of data is generated during patient care, but systematically accessing and analysing this information has always been a challenge, with much of it being wasted and never shared widely ^2^. A general underreporting of research and data in the medical literature is partly to blame for this waste as much good-quality science, including negative results or small studies, are never published ^3– 5^. Even those findings that do get published can often only be shared after a lengthy publishing process; the need for faster access to research findings is particularly evident in public health crises ^6^ but is pertinent to acute care in general.

By launching a new channel in *F1000Research* that focuses on all areas of critical care, we offer the community a new platform for sharing new research and debate without barriers, making it easier for busy clinicians and researchers to write up their findings quickly and make them available, ultimately helping other physicians improve the care of patients with life-threatening injuries and illnesses.

*F1000Research* operates a unique post-publication peer review model that allows authors to take charge of their own publications: Submitted articles (Research, Methods, Clinical Practice Articles and so on) are published quickly once they have passed a basic suitability check, which covers issues such as ethical approval. Peer review by invited experts takes place openly after publication, with referees being named and their reports being published. Authors can address any criticisms by publishing a revised version, and all articles that pass peer review are listed in PubMed and other bibliographic databases.

The Critical Care channel welcomes research across the broad field of acute care and emergency medicine, including all aspects of critical cardiovascular, respiratory, renal and gastro-intestinal problems, neurological and metabolic complications, pediatric critical care, sepsis and multiple organ failure.

Research into, and discussions of, ethical and organisational challenges in critical care, as well as advances in techniques and equipment are also encouraged. In order to address the current publication bias towards ‘exciting’ and ‘positive’ research, we welcome confirmatory studies or negative results, as long as they are supported with original data and sufficient methodological details that make it possible for others to repeat the analysis if they wish.

As a special service to the community, the Critical Care channel includes short F1000 Faculty Critiques, which are commissioned from F1000 Faculty Members, who evaluate the current evidence-based literature with the aim to help readers understand whether a specific recent study is likely to change clinical practice.

Finally, an archive of posters and slides that were presented at the various annual International Symposia on Intensive Care and Emergency Medicine ( ISICEM) is also available as part of the channel.

I hope that the channel will create lively debate and entice you to share your research and insights – big and small – so the critical care community can benefit from your experience!
